# Preferential suppression of *Anopheles gambiae* host sequences allows detection of the mosquito eukaryotic microbiome

**DOI:** 10.1038/s41598-017-03487-1

**Published:** 2017-06-12

**Authors:** Eugeni Belda, Boubacar Coulibaly, Abdrahamane Fofana, Abdoul H. Beavogui, Sekou F. Traore, Daryl M. Gohl, Kenneth D. Vernick, Michelle M. Riehle

**Affiliations:** 1 Department of Parasites and Insect Vectors, Unit of Genetics and Genomics of Insect Vectors, Institut Pasteur, Paris, France; 2 CNRS Unit of Hosts, Vectors and Pathogens (URA3012), Paris, France; 3 Malaria Research and Training Centre (MRTC), Faculty of Medicine and Dentistry, University of Mali, Bamako, Mali; 4 Centre de Formation et de Recherche en Santé Rurale de Mafèrinyah, Conakry, Guinea; 5 University of Minnesota Genomics Center, Minneapolis, Minnesota USA; 6 Department of Microbiology and Immunology, University of Minnesota, Minneapolis, MN USA

## Abstract

*Anopheles* mosquitoes are vectors of the human malaria parasite, *Plasmodium falciparum*. The vector microbiota is a likely factor influencing parasite transmission. The prokaryotic microbiota of mosquitoes is efficiently surveyed by sequencing of hypervariable regions of the 16s ribosomal RNA (rRNA) gene. However, identification of the eukaryotic microbiota by targeting the 18s rRNA gene is challenging due to simultaneous amplification of the abundant 18s rRNA gene target in the mosquito host. Consequently, the eukaryotic microbial diversity of mosquitoes is vastly underexplored. An efficient methodology is needed to identify this component of the microbiota, expected to include relatives of *Plasmodium*. Here, we use defined panels of *Anopheles* samples from West Africa to test two experimental PCR clamp approaches to maximize the specific amplification of 18s rRNA gene hypervariable regions from eukaryotic microbes: anneal-inhibiting blocking primers and peptide-nucleic acid (PNA) oligonucleotide blockers. Of the two, PNA blockers were the only efficient blocking strategy, allowing a reduction of mosquito 18s rRNA gene sequences by more than 80% for the V4 hypervariable region. These PNA blockers will facilitate taxonomic profiling of the eukaryotic microbiota of the *A*. *gambiae* species complex, and contribute to a better understanding of microbial influence upon immunity and pathogen infection.

## Introduction

Much of the eukaryotic microbial diversity in natural environments remains to be characterized, despite the critical importance of these lineages as pathogens, symbionts and commensals, environmental quality indicators, and markers of past environmental changes. Mosquito vectors of malaria and other pathogens are exposed to microbes at every developmental stage, and microbes can also be vertically transmitted from mother to offspring^1^. The mosquito larval pool is a microbial soup, likely imposing continual pathogen pressure on the aquatic larvae. In addition, blood feeding adult females are exposed to blood-borne pathogens such as *Plasmodium*, and adult males and females are exposed to plant-borne microbes during nectar feeding. Exposure of mosquitoes and their ancestors to the numerous eukaryotic and prokaryotic microbes encountered in the aquatic environment, which include aquatic relatives of *Plasmodium* within the apicomplexan lineage, has probably been integral to shaping the evolution of the mosquito innate immune system.

Mosquitoes of the *Anopheles gambiae* species complex are primary African vectors of the human malaria parasite, *Plasmodium falciparum*, which is responsible for extensive human morbidity and mortality. The most important vector species in the complex are *A*. *gambiae* and its sister taxon *A*. *coluzzii*, previously called the *A*. *gambiae* S and M molecular forms, respectively^2^. The two species occupy different larval ecological niches, and are probably adapted to distinct microbial communities^3–5^. Multiple studies have catalogued the prokaryotic microbiota composition of different *Anopheles* mosquito populations by sequencing of 16s rRNA hypervariable regions^6–13^. However, the eukaryotic members of the *Anopheles* microbiota remain poorly described, largely due to the absence of high-throughput methods for their identification.

Microscopic examination and insect pathology studies have identified a handful of eukaryotic microbes, including microsporidia, gregarines and trypanosomatids, in different mosquitoes^14–24^. Little attention has been paid to *Anopheles* vectors of malaria in these manual explorations of individual microbe taxa. A bulk deep sequencing study of arthropod fauna identified some eukaryotic microbes, but these were incidental to the main focus on arthropod phylogenies^25^. To our knowledge, a comprehensive metagenomic catalogue of the eukaryotic microbiome has not been reported from any insect. The focus of the current work is the development of methodological approaches to comprehensively characterize the *Anopheles* eukaryotic microbiome.

*Anopheles* mosquitoes mount a functional immune response during infection with *Plasmodium*. The midgut bacterial flora is antagonistic for *Plasmodium* infection, probably mediated by at least activation of basal immunity^26, 27^. Bacteria may also influence malaria infection outcome by production of reactive oxygen species that directly target *Plasmodium* parasites in the midgut^28^, or by secreted factors that compromise both mosquito and *Plasmodium* survival^29^. A correlation was found between components of the midgut microflora and *Plasmodium* infection status in mosquitoes from Cameroon^30^.

A small number of functional studies have demonstrated interactions between eukaryotic microbes and mosquito immunity. The yeast *Wickerhamomyces* produces a toxin with antimicrobial, including anti-*Plasmodium*, activity *in vitro* ^31, 32^. Co-infection of mosquitoes with the microsporidian *Vavraia* can inhibit development of rodent malaria parasites^33^. Finally, it was proposed that a gregarine protozoan could harbour chikungunya virus, and help maintain the virus in the *Aedes* mosquito vector population^34^.

A major obstacle in high-throughput surveys of eukaryotic microbiota in eukaryotic hosts is that the host organism has its own 18s rRNA gene, and general amplification of this host gene overwhelms the microbial 18s rRNA gene signal. To detect eukaryotic microbes, an efficient method is essential to preferentially block host 18s rRNA gene amplification, while simultaneously allowing robust amplification of the microbial 18s rRNA gene target. Two main PCR clamping strategies have been proposed to uncover the eukaryotic microbial signal by preferentially inhibiting PCR amplification of the overwhelming molar excess of host background target. First, anneal-inhibiting blocking primers (here called annealing blockers) bind to PCR primer annealing sites and competitively inhibit annealing and subsequent extension by the PCR primers. Annealing blockers were reported to decrease amplification of host background target DNA^35^. Second, Peptide-Nucleic Acid (PNA) oligonucleotides (here called PNA blockers) bind within the target amplicon and biochemically inhibit extension of the non-desired products. PNA blockers were used to decrease amplification of mitochondrial and plastid 16s rRNA gene target^36^. Here we evaluate annealing blockers and PNA blockers for the metagenomic identification of eukaryotic microbes associated with African environmental samples of *A*. *gambiae*.

## Results

### *In-silico* prediction of annealing blocker and PNA blocker specificity

**Figure 1:**
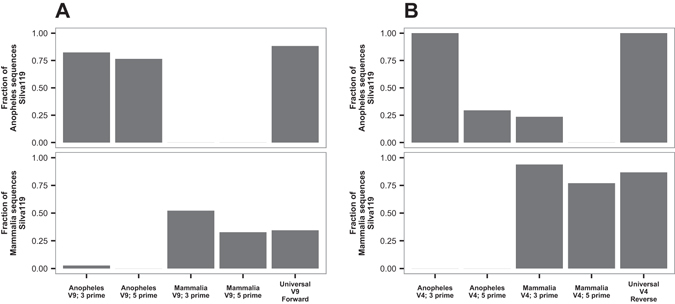
*In silico* evaluation of target specificity for the annealing blockers of *Anopheles* and *Mammalia*. The y-axis represents the relative fraction of 18s rRNA gene sequences present in version 119 of the Silva database that match the primer indicated on the x axis, with match defined as a weighted score <1.0 (See Methods). (**A**) Specificity for V9 region annealing blockers. (**B**) As in A, but for the V4 region annealing blockers. As positive controls, the reference universal eukaryotic PCR amplification primers V9-Forward and V4-Reverse primers were also queried against the Silva database (right bar in each frame).

Annealing blockers were designed as dual priming oligonucleotides with the 5′ segment overlapping the universal amplification primer and the 3′ segment specific to the non-desired 18s rRNA gene sequence. For the V4 hypervariable region, annealing blockers were designed that separately target *A*. *coluzzii* and mammal sequences respectively, whereas for the V9 region, an annealing blocker was designed targeting *A*. *coluzzii*, while for mammalian sequences the mammal blocker of the Earth Microbiome Project (EMP) was used. The blockers were evaluated *in silico* to determine the fraction of matching 18s rRNA gene sequences among *Anopheles* and *Mammalia* references in the Silva 119 database (match was defined as a weighted score <1.0, see Methods). For the V9 region blocker (Fig. 1A), both segments matched greater than 75% of *Anopheles* 18s rRNA gene sequences, a level similar to that of the V9-Forward universal eukaryotic amplification primer. In contrast, the 3′ segment of the EMP mammal blocker matched at most 50% of the mammalian sequences from the Silva 119 database. In the V4 region (Fig. 1B), the mammal and *Anopheles* blockers matched most of their reference sequences from the Silva 119 database, except for the 5′ segment of *Anopheles* blocker, which matched only 29% of *Anopheles* reference sequences in Silva 119, all of them corresponding to *A*. *gambiae*.

**Figure 2:**
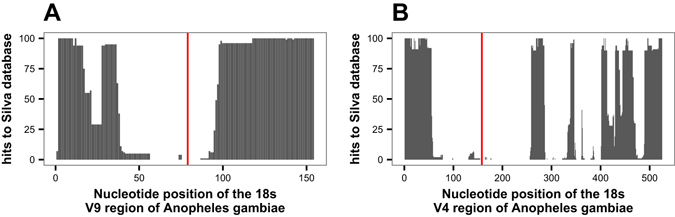
Identification of PNA oligonucleotide blocker candidates for the V9 region of *Anopheles*. In order to design PNA blockers specific to *Anopheles*, but allowing efficient amplification of other eukaryotic organisms, the Silva eukaryotic database was queried with mosquito candidate sequences to identify those with the smallest number of non-mosquito hits. (**A**) The full-length sequence of the V9 hypervariable region of *A*. *gambiae* was split *in silico* into all possible 17mers, and each 17mer was queried against the full length 18s rRNA gene sequences from the Silva 119 database for all non-mosquito eukaryotes. The number of matches was graphed (y-axis), limiting the representation to a maximum of 100 hits. The location of the selected current PNA blockers is indicated (red vertical line). (**B**) As in A, but for V4 region PNA oligonucleotide candidates.

PNA blocker design was carried out by mapping all possible 17 mers contained in the reference sequence of the hypervariable regions V4 and V9 of *A*. *gambiae* 18s rRNA gene against the full set of 18s rRNA gene sequences from the Silva database version 119, allowing at most one mismatch. The final PNA blockers AgV4-PNA and AgV9-PNA were selected from among the 17 kmers that were present exclusively in *Anopheles* sequences, thus displaying maximum specificity (Fig. 2).

### Measurement of annealing blocker and PNA blocker efficiency by high-throughput sequencing

A defined test panel representing three biological conditions was constructed by pooling characterized wild *A*. *coluzzii* samples of i) mature fourth-instar larvae, ii) adult female mosquitoes, all containing exclusively human bloodmeals, and all positive for presence of *P*. *falciparum* parasites, as determined independently by molecular diagnostic typing, iii) adult female mosquitoes, all containing exclusively animal bloodmeals, as determined independently by molecular diagnostic typing. The mosquito bloodmeals were obtained by wild mosquito feeding in nature. Specific molecular diagnostic typing of the sample DNA was used for animal blood source and presence of *Plasmodium* parasites (see Methods for details on molecular typing). Thus, the test panel is comprised of pooled samples with known attributes, and serves as a series of positive and negative controls for the different conditions, but also captures the natural variation of wild-collected samples. The efficiency of inhibition by annealing and PNA blockers was empirically evaluated using DNA from the three samples of the test panel. For each sample, the V4 or the V9 hypervariable region of the 18s rRNA gene was amplified with universal PCR primers i) without including any blocker, ii) with the designed annealing blockers for mammal and *Anopheles* in equal amounts, and iii) with the designed PNA blocker for *Anopheles* at different concentrations. The resulting amplicons were sequenced, and the blocking efficiency of both kinds of blockers was determined as the fraction of residual *Anopheles* (host background) amplicons obtained in each sample and blocking condition.

A total of 5,052,260 paired-end MiSeq reads were generated for V4 amplicons, with an average of 157,883.125 paired-ends reads per sample after the demultiplexing step. Of these, 4,137,310 (81.89% of the total) full-length V4 amplicons were reconstructed after quality filtering and joining steps (129,290.94 amplicons per sample on average), and clustered into 119 Operational Taxonomic Units (OTUs) of a minimum size of 5 reads from which the taxonomic profiles of the samples across all conditions (with/without PNA blockers or PCR annealing blockers) were determined by sequence similarity against the 18s rRNA gene subdivision of Silva database version 119.

**Figure 3:**
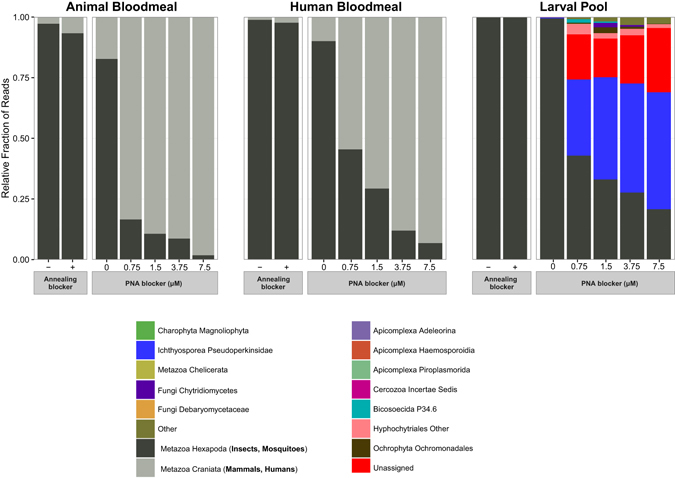
Taxonomic profile of 18s rRNA gene amplicons for the V4 hypervariable region generated under different blocking conditions. Mammalian and mosquito hits are shown in grey scale while those of eukaryotic microbes are shown in colour. In all three samples, use of the mosquito specific PNA blocker significantly decreased mosquito reads. In animal and human bloodmeal samples with PNA blockers, human and mammal reads were predominant, whereas in the larval pool sample, eukaryotic microbes represented the majority of sequence reads when PNA blockers were used. For simplicity, sequences were collapsed at level 6 of the Silva 119 eukaryotic taxonomy, and lineages with less than 100 total amplicons were collapsed into the *Other* class (12 lineages for the V4 hypervariable region; 23 lineages for the V4 hypervariable region) to facilitate legend visualization.

As expected, mosquito was the predominant taxonomic group in V4 amplicons from samples amplified without any blocker, ranging from >99% of the amplicons in larval samples to 80–97% of the amplicons in human and animal fed samples (Fig. 3). The lower percentage of mosquito reads in human and animal fed samples is a consequence of the amplification of sequences derived from the bloodmeal. The efficiency of PCR annealing blockers was negligible, with the corresponding samples showing a taxonomic profile similar to the samples without any blocker, dominated by mosquito sequences. The annealing blocking primers did shift the reaction threshold cycle (Ct), indicating interference with PCR amplification, but data below indicate that this effect was probably non-specific. As a general rule, fairly permissive hybridization conditions are preferred for microbiome profiling, so increasing PCR stringency in an effort to improve annealing blocker performance would in turn hinder the ability to achieve high resolution of the eukaryotic microbiome. Attempts to amplify with annealing blockers using the proofreading enzyme, KAPA polymerase, as used for PNA blockers, yielded no amplification, likely due to interference of the annealing blocker C3 spacer region with KAPA amplification by an unknown mechanism. The lack of ability to use KAPA proofreading polymerase is another argument against annealing blockers, because use of a proofreading polymerase can increase information content by amplifying target from organisms with primer mismatches^37^.

In contrast, the *Anopheles* V4 PNA blocker displayed high blocking efficiency of *Anopheles* host 18s rRNA sequences in all three samples (larval, human-bloodmeal containing adult, and animal-bloodmeal containing adult), with a PNA concentration-dependent reduction of mosquito host sequences (Fig. 3). In the case of human and animal blood-containing adult samples, the decrease in mosquito reads is paralleled by an increase in mammalian reads, pointing to the possibility of using a second mammal PNA blocker in order to analyse samples containing human or animal 18s rRNA gene targets. In contrast, in the larval sample, a similar decrease of mosquito reads in PNA blocker samples is instead correlated with an increase in reads from microbial eukaryotic lineages (Fig. 3). For example, reads belonging to the *Pseudoperkinsidae* lineage of protist parasites^38^ increased to 48.2% of the amplicons in the larval sample with 7.5 μM PNA, as compared to less than 1% of the amplicons without PNA blocker.

The second most abundant group in the PNA-treated larval sample corresponds to Unassigned amplicons, with no taxonomic assignment by sequence similarity against the Silva 119 database. These Unassigned amplicons, 26% of sequences in the larval sample with 7.5 μM PNA, indicate the limitation of current reference databases, and highlight the need for additional phylogenetic placement methods for more precise classification^39^.

**Figure 4:**
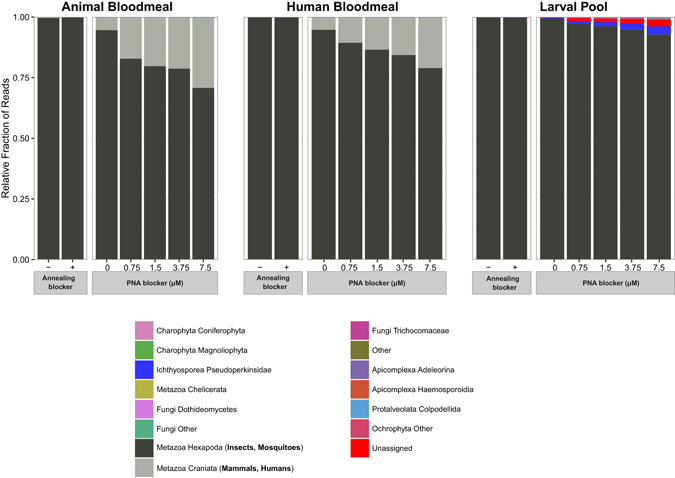
Taxonomic profile of 18s rRNA gene amplicons for the V9 hypervariable region generated under different blocking conditions. Mammalian and mosquito hits are shown in grey scale while those of eukaryotic microbes are shown in colour. In all three samples, use of the mosquito specific PNA blocker decreased mosquito reads, though to a lesser extent than with the V4 PNA blocker (compare to Fig. 3). Sequences were collapsed at the V6 level of Silva 119 eukaryotic taxonomy, and lineages with less than 100 total amplicons were collapsed into the *Other* class to facilitate legend visualization.

The results for the V9 hypervariable region were similar (Fig. 4). In this case, a total of 5,422,957 paired-end MiSeq reads were generated, with an average of 169,467.4 reads per sample after the demultiplexing step. Of these, 4,988,634 (91.99% of the total) full-length V9 amplicons were reconstructed after quality filtering and joining steps (155,894.81 amplicons per sample on average), and these were clustered into 134 OTUs of a minimum size of 5 reads. Similar to the V4 amplicons, the blocking efficiency of *Anopheles* and mammal annealing blockers was negligible, with the corresponding samples showing similar taxonomic profile as reference samples without any blocker. A PNA concentration-dependent reduction in mosquito amplification with increasing *Anopheles* V9 PNA blocker concentrations was observed, as seen also for the V4 PNA blocker, although the concentration-dependent effect on background reduction by the V9 PNA blocker was less efficient than by the V4 PNA blocker.

**Figure 5:**
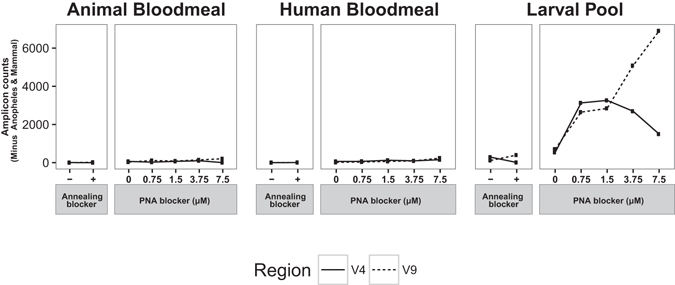
Efficiency of 18s rRNA gene blocking strategies for detection of the *Anopheles* eukaryotic microbiome. *Anopheles* and mammal 18s rRNA gene sequences were removed, and the numbers of 18s rRNA gene amplicons for the V4 (solid line) and V9 (dashed line) regions are indicated. The amplicon counts of non-*Anopheles* and mammal sequences indicates the efficiency of detection of eukaryotic microbiome lineages, shown as a function of blocking strategy used as well as PNA blocker concentration.

As with V4, the decrease in the fraction of mosquito reads in human and animal-bloodmeal containing adult samples is accompanied by an increase in the fraction of mammalian reads (Fig. 5). The decrease in the fraction of mosquito reads in larval samples is accompanied by an increase in the fraction of *Pseudoperkinsidae* and Unassigned reads, although to a lesser extent than for V4 amplicons (3.34% Pseudoperkinsidae and 2.7% Unassigned of V9 amplicons with 7.5 μM PNA, respectively). When sequences originating from mosquito and mammals are removed from the analysis, only larval samples display an important increase in amplification of eukaryotic microbial lineages due to PNA blocking. Larval samples with PNA blocker exhibited efficient blocking of mosquito host 18s rRNA gene amplification, increasing the amplification of the eukaryotic microbiome, with the V9 region showing the maximum number of eukaryotic microbiota reads at 7.5 μM PNA concentration whereas the V4 region displayed maximum eukaryotic microbiota reads at 1.5 μM PNA concentration (Fig. 5).

### PNA blocker-dependent identification of *Plasmodium* in human-bloodmeal containing adult samples

**Figure 6:**
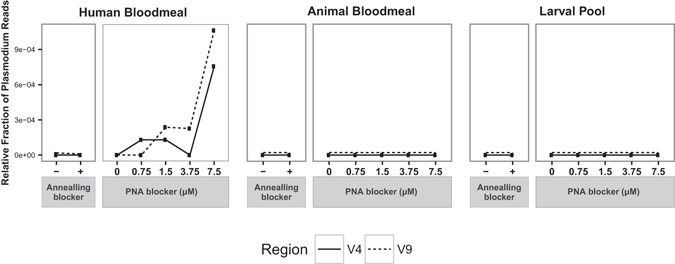
Positive control test of blocking strategies for detection of a known eukaryotic microbe. Relative fraction of 18s rRNA gene amplicons from the V4 (solid line) and V9 (dashed line) hypervariable regions assigned to the *Plasmodium* genus in the three samples under the different blocking conditions. All human bloodmeal-containing adult mosquito samples were positive for the presence of *P*. *falciparum* by molecular diagnostic assay. Dashed lines for Animal Bloodmeal and Larval Pool samples were superimposed with solid lines, and are shown offset to improve visibility.

The human-bloodmeal containing adult mosquitoes pooled to create the current test sample were all independently confirmed by molecular diagnostic typing to be positive for *Plasmodium*, so the distribution of *Plasmodium* sequences across these samples is a positive control for sensitivity and specificity of PNA blocking. Compared across the different samples, *Plasmodium* sequences were detected exclusively in human-bloodmeal containing adult samples treated with PNA blocker (Fig. 6). No *Plasmodium* reads were detected in any of the other samples. Notably, *Plasmodium* reads were not detected in the *Plasmodium*-positive (human bloodmeal-containing adult) sample without PNA blocker, due to the massive swamping of the *Plasmodium* target by amplification of mosquito 18s rRNA gene target in absence of PNA blocker. In addition, a PNA-concentration dependent increase in the fraction of *Plasmodium* sequences was observed, with maximal detection of *Plasmodium* in the human bloodmeal-containing adult sample at 7.5 μM PNA concentration for both the V4 and V9 amplicons (Fig. 6). The irregular appearance of the 3.75 μM PNA point in the human bloodmeal sample suggests there may have been an aberration or artefact, which was nevertheless not a general case for the 3.75 μM PNA blocker (compare with Fig. 5, larval pool). These results confirm the designed PNA blockers as a valid strategy for high-throughput discovery of eukaryotic microbes in 18s rRNA gene metagenomic surveys of *Anopheles* larval and adult mosquitoes.

### PNA-dependent increase of species richness and evenness

**Figure 7:**
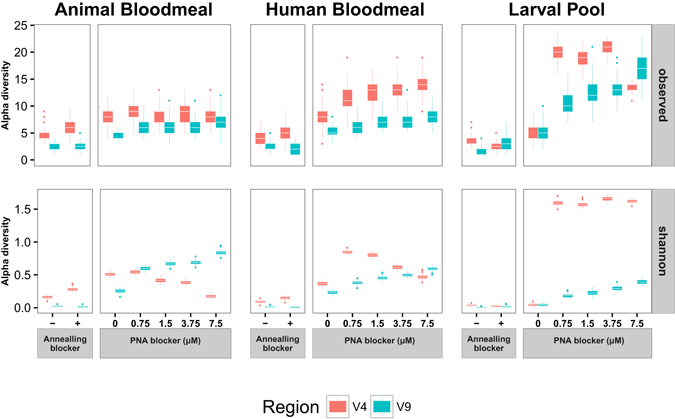
Influence of 18s rRNA gene blocking strategies for taxonomic richness and evenness. Alpha-diversity boxplots for the three samples of the analysis panel under different amplification conditions in the V4 (orange) and V9 (blue) hypervariable regions. Each boxplot represents 100 estimates of alpha-diversity (richness: observed species, top plot; and evenness: Shannon index, bottom plot) computed from 100 random subsamples of size 1000 in each amplicon dataset. Whiskers represent values within 1.5x of the inter-quartile range, from the third (upper whisker) and first (lower whisker) quartiles of the alpha-diversity distribution. Outlier values beyond the end of the whiskers are plotted as points.

Without efficient host blockers (either annealing or PNA), the massive amplification of host DNA in 18s barcoding surveys hides the eukaryotic microbiome, because only host 18s DNA is amplified. An efficient blocking strategy should decrease the amplification of host DNA, allowing the detection of eukaryotic microbial organisms, with an expected increase in the alpha diversity of the samples. Consistent with this hypothesis, PNA treatment increases both the richness (measured as numbers of observed OTUs) and evenness (measured as the Shannon diversity index) in larval and adult samples of the defined control sample panel. In contrast, annealing blockers have little or no effect on either of these alpha-diversity metrics due to their inefficient blocking (Fig. 7). The greatest increase in diversity occurs following PNA treatment, and is observed in the evenness of larval samples. Minimal evenness is seen in the absence of PNA blocker (that is, >99% of the sequences correspond to host DNA), while maximal evenness is detected in V4-region amplicons in which host DNA comprises less than 50% of the total sequences (Fig. 7). In human bloodmeal and animal bloodmeal samples, the progressive replacement of mosquito host by mammal sequences with increasing PNA concentration explains the patterns of evenness observed.

Rarefaction analyses of V9 and V4 amplicon datasets yields similar results, showing that the eukaryotic community found in larval samples amplified with PNA blockers is the most phylogenetically diverse of the three sources of samples analysed (Figure S1). However, the presence of PNA blocker does not affect the relative fractions of individual OTUs and other taxonomic subdivisions of the eukaryotic microbial community recovered in larval samples (chi-square p = 0.92 for V4 and p = 1 for V9, Figure S2). These results indicate that the increase in evenness due to PNA blocker observed in the alpha diversity analysis is the consequence of the efficient blocking of mosquito host 18s rRNA gene amplification, allowing emergence of the eukaryotic microbial community signal, without effect on the relative proportions of the lineages in the larval community.

### PNA blocking in other *Anopheles* species

**Figure 8:**
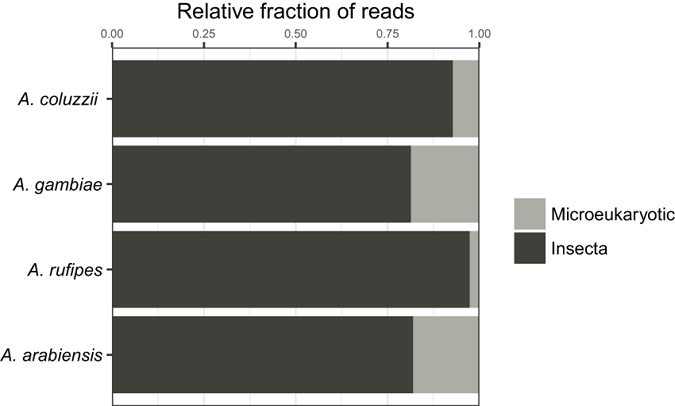
PNA blocking efficiency across multiple *Anopheles* species. Proportions of sequence reads assigned to insects (dark grey) and eukaryotic microbes (light grey) are showing 4 Anopheline species. Approximate evolutionary divergences^40^ of each species from *A*. *coluzzii* are, *A*. *gambiae*, 0.5 million years; *A*. *arabiensis*, 1.8 million years*;* and *A*. *rufipes*, >20 million years.

To determine the generality of these tested PNA blockers for use in species other than *A*. *coluzzii*, we empirically tested blocking efficiency for the V9 region of the 18S rRNA gene using test sets of larval samples for three other *Anopheles* species (Fig. 8). Two of these species are also within the *A*. *gambiae* species complex (*A*. *gambiae* and *A*. *arabiensis)*, and the third species, *A*. *rufipes*, is outside of the *A*. *gambiae* species complex. Evolutionary divergence from *A*. *coluzzii* is approximately 0.5 million years for *A*. *gambiae*, 1.8 million years for *A*. *arabiensis*, and more than 20 million years for *A*. *rufipes* ^40^.

While the degree of observed PNA blocking efficiency was variable across species, in all cases the blocking was efficient enough to yield sequence reads for assessment of the eukaryotic microbiome on a comparable level as the blocking observed for *A*. *coluzzii* (Fig. 8). Thus, the PNA blocker AgV9-PNA can be expected to efficiently block host 18s rRNA gene amplification from both closely related as well as more distant *Anopheles* taxa.

## Discussion

Although the bacterial component of the microbiome has been the focus of most metagenomic research to date, prokaryotes, viruses, and eukaryotic microbes together form a complex network of ecological interactions that ultimately defines the complete microbiome. Here we focused on improving the detection and cataloguing of the essentially unexplored insect eukaryotic microbiome. We provide an efficient strategy for high-throughput characterization of the eukaryotic flora of *Anopheles* mosquitoes using PNA blockers targeting V4 and V9 hypervariable regions of the mosquito 18s rRNA gene. Here, the strategy was developed and validated using a control panel of defined, pooled samples. The strategy could subsequently be applied to field collected samples of different developmental stages, with metadata for ecological zone, mosquito species, and larval pool type.

The V4-region PNA blocker displays the highest blocking efficiency, reducing the fraction of host DNA amplification by 46.0x in the animal-fed adult mosquito sample (from 82.8% to 1.8% of mosquito amplicons at 7.5 μM concentration of V4-PNA blocker) and by 4.8x in the larval sample (from 99.5% to 20.8% of mosquito amplicons at 7.5 μM concentration of the PNA blocker). The efficiency of the V9 PNA blocker is lower in comparison, probably due to the smaller size of the V9 region, leading to higher amplification of host DNA. However, in both cases amplification of the eukaryotic microbiome was substantially increased in the larval sample, as evidenced by the sequencing depth of the samples after filtering out mammalian and insect sequences. There are slight differences in the OTU catalogues generated from the two different amplicons (see Figs 3 and 4), which is likely the result of the smaller V9 amplicon (153 bp on average) providing less information content than the longer V4 amplicon (409 bp on average), perhaps combined with the relative immaturity of the databases.

This decrease in host DNA amplification leads to an increase of the diversity and richness of the larval sample, as a consequence of the amplification of eukaryotic lineages that constitutes the eukaryotic microbiome. This consistency in microbial composition across PNA concentrations is in accord with the reported use of PNA blockers for mitochondrial and plastid 16s rRNA genes in plant root and soil samples^36^. The eukaryotic microbiome of larval samples includes an important component of ichthyosporeans of the Pseudoperkinsus group, a lineage of unicellular organisms that includes parasites and commensals of invertebrates, fish, amphibians, birds and mammals^41^. This group has been placed at the boundary between animals and fungi^42^, and new species have been discovered through examination of digestive tracts of arthropods and other groups of invertebrates^41^.

Recently, a phylogenetic analysis including all complete 18s rRNA gene sequences available in GenBank revealed a large and previously hidden diversity of non-fungal unicellular opisthokonts including ichthyosporeans^43^. More data from field samples would be necessary to evaluate the presence of this group in natural mosquito populations, but the predominance in our larval sample points to these taxa as a likely important component of the mosquito eukaryotic microbiome. Similarly, the large fraction of Unassigned sequences observed in the 7.5 μM PNA larval sample is consistent with other surveys of eukaryotic microbial environmental diversity based on 18s rRNA gene sequences, where a large fraction of observed sequences could not be assigned to any described species, suggesting in some cases even the presence of new eukaryotic kingdoms^44, 45^. The density of Unassigned eukaryotic sequences revealed by the PNA blocking strategy presented here is promising for future work on the environmental ecology of eukaryotic microbial diversity in mosquito vectors.

In contrast with the results observed in the larval samples, blood-fed adult mosquito samples would require a second PNA blocker specific for mammals to increase the amplification of the eukaryotic microbiome. Such blockers can be designed, but would need to be specifically directed against human and the few common mammals in a zone because of the high target specificity of PNA blockers^46^. With the use of PNA blockers, *Plasmodium* 18s rRNA gene sequences can be detected in human-fed adult mosquitoes, while they are undetectable without the blockers. These results indicate that the reported PNA blockers could be implemented in high-throughput field surveys of adult mosquito populations to simultaneously explore the presence and diversity of *Plasmodium* and other related apicomplexan parasites in adult mosquito populations.

High throughput surveys of eukaryotic microbial diversity by barcoding 18s rRNA gene sequences has been carried out in natural environments^44, 45, 47–49^, confirming that the evolutionary and ecological importance of eukaryotic microbes is much higher than traditionally thought and suggesting that the number of eukaryotic microbial species may easily exceed one million, with a large fraction of “unknown” eukaryotic microbial diversity that remains to be characterized^50^. However, characterizing eukaryotic microbial communities in eukaryotic hosts faces the technical obstacle of the presence of the conserved 18s rRNA gene in both microbiome and host. This requires strategies to preferentially detect the microbial components, which was the focus of the present study.

To overcome lack of knowledge in eukaryotic microbial diversity, the Consortium for the Barcode of Life constituted in 2012 the Protist Working Group (ProWG), whose main objective is to establish universal criteria for barcode-based species identification in eukaryotic microbes^46^. This ProWG proposes a two-step approach, comprising a preliminary identification using a universal eukaryotic barcode, called the pre-barcode, followed by a species-level assignment using different group-specific barcodes^51^, and proposes the V4 hypervariable region of the 18s rRNA gene as universal eukaryotic marker for the pre-barcoding step. Here, we found that amplification and sequencing of the V4 region using our PNA blockers is the most informative approach for discovery of the *Anopheles* eukaryotic microbiome.

The present work opens the way towards the high-throughput characterization of the eukaryotic microbial diversity associated with the *Anopheles* microbiome, which will also facilitate study of the interactions between prokaryotic and eukaryotic components of mosquito microbiome in the context of vector competence for *Plasmodium* parasite transmission. This approach represents a significant advance for generating a complete picture of the microbiota encountered in the *Anopheles* mosquito, the vector of human malaria.

## Methods

### Blocking primer design

The annealing blocker primer strategy is based on methods^35^ that were also used by the Earth Microbiome Project to design a mammal-blocking primer (*Mammal_block_I-short_1391f*) for the V9 region of the 18s rRNA gene (http://www.earthmicrobiome.org/emp-standard-protocols/18s/). These annealing blockers are dual priming oligonucleotides consisting of two separate priming regions joined by a polydeoxyinosine linker. The 5′ segment is longer (18–25 nt) and binds to the same region as one of the universal amplification primers, whereas the 3′ segment is shorter (6–12 nt), selectively binds to the non-desired target sequence, and ends with a chemical modification (C3 spacer) that prevents extension during the PCR reaction of the non-desired target^52^. Based on these considerations, an *Anopheles* blocking primer for the V9 region of the 18s rRNA gene (*Anoph_V9_block*) was designed covering the same region as the *Mammal_block_I-short_1391f* blocker (overlapping with the universal V9 forward primer), with the 5′ segment identical to that of *Mammal_block_I-short_1391f* followed by five deoxyinosine (polyI region) molecules and a 3′ segment specific to *Anopheles* (non-desired target) 18s rRNA gene sequence (Figure S3). Blocking primers for *Anopheles* (*Anoph_V4_block*) and mammals (*Mammal_V4_block*) targeting the V4 region of the 18s rRNA gene were designed in a similar way, in this case overlapping with the universal V4 reverse primer (Figure S4).

The specificity of the 5′ and 3′ segments of all blocking primers was evaluated with the analyze_primers.py program of the PrimerProspector package^53^ against reference 18s rRNA gene sequences from version 119 of the Silva database^54^. This program computes a local alignment for the input primer(s) against target sequences and determines a weighted score based on the number of mismatches and gaps, giving large penalties to gaps and mismatches at the 3′ end of the primer. The program taxonomic_coverage.py of the PrimerProspector package was used to extract the fraction of *Anopheles* and mammal sequences in Silva 119 database matched by each blocking primer segment (with match defined as a weighted score < 1.0). The sequences of all oligonucleotides used in the current study are given in Table S1.

### Data availability

Data are available in the EBI European Nucleotide Archive under study accession number PRJEB20080, at the URL http://www.ebi.ac.uk/ena/data/view/PRJEB20080.

### Peptide nucleic acid (PNA) design

Candidate PNA sequences for blocking *A*. *gambiae* 18s amplification were identified using a described methodology^36^. We *in-silico* fragmented the sequences for two hypervariable regions of the 18s rRNA gene of *A*. *gambiae*, V4 (541 nt; extracted from SilvaID: AAAB01003942) and V9 (170 nt; extracted from SilvaID: AAAB01003343), into 16 nt-overlapping k-mers of 17 nt length using the *splitter* program from the EMBOSS package^55^. We queried the resulting kmers for matches against the representative set of 18s rRNA gene sequences from the Silva database version 119 using bowtie^56^ allowing at most one mismatch (~6%), which is the described threshold for efficient amplification blocking^46^. PNA sequence candidates targeting V4 or V9 hypervariable regions were identified from the set of *A*. *gambiae*-specific kmers according to bowtie alignments.

The previous species taxon, *A*. *gambiae*, was recently divided into the species *A*. *gambiae* and *A*. *coluzzii* ^2^. The Silva 119 database 18s rRNA gene entry for the taxon is currently still referenced as *A*. *gambiae*, without distinguishing between the two new species. To confirm the applicability of the Silva database entry for both species, we used vcf files from the *Anopheles gambiae* 1000 Genomes Consortium project^57^ to extract the V4 and V9 region from whole-genome sequences of both *A*. *gambiae* and *A*. *coluzzii* individuals, and sequences were aligned using Jalview (http://www.jalview.org/). Identity over the current PNA oligonucleotide regions was 100% between *A*. *gambiae* and *A*. *coluzzii* (Figure S5, V9 region; Figure S6, V4 region). Consequently, the current PNA oligonucleotides are equally applicable to both species.

Melting temperature, GC content, and potential hairpin structures for candidate PNAs were evaluated with the PNA tool of PNABio (CA, USA) (http://pnabio.com/support/PNA_Tool.htm). Candidate 17 kmers were filtered for hairpin structures, a purine content below 50% and a melting temperature Tm of 76–79 °C. The final anti-*A*. *gambiae* V9 PNA (AgV9-PNA) 5′-CCGTGCCAACTGCAAC-3′ and V4 PNA (AgV4-PNA) 5′-ACGCCCAGGTACACC-3′ were synthesized by PNA Bio (CA, USA). Lyophilized PNAs were spun down and resuspended in an appropriate volume of water to make 100 μM stock solutions.

### Mosquito DNA extraction and quantification

Collection of mosquito samples in Takan, a village in Southern Mali, West Africa, was previously described^58^. Permission was obtained from village authorities to collect mosquitoes in the villages. Mosquito DNAs were prepared using DNAzol as described^58^. All DNA extractions were done on whole mosquito carcasses, either adults or larvae. For each control sample, ten individual mosquito DNA samples were pooled at equal volume and a total of control samples were made representing three distinct mosquito life stages i) larvae, ii) female adults containing human bloodmeal, (mosquitoes that fed in nature with blood confirmed as human derived, all positive for presence of *Plasmodium*)^58^, and iii) female adults containing animal bloodmeal (as with ii except the bloodmeal was of animal origin, and was negative for *Plasmodium*). Methods for detection of bloodmeal and Plasmodium are PCR based and described in detail elsewhere^58^.

The control samples were artificially constructed as an informative technical reagent for the testing and evaluating blocking strategies, and not for biological discovery of microbial taxa. In order to be biologically informative, subsequent work using the methodology developed here will require unbiased sampling. The human fed, *Plasmodium*-positive pool was used as a positive control for the detection of the known microbial eukaryote, *P*. *falciparum*. Metagenomic sequencing would not be cost effective as a method of detection for *Plasmodium*-infected wild mosquitoes. Moreover, screening for *Plasmodium* in pooled samples would have low information value because a pool with a single highly infected individual could not be distinguished from a large number of individuals with low infection.

To assess the efficiency of blocking in other mosquito species, we amplified and sequenced 18S rRNA gene V9 variable region amplicons from larval test sets of *A*. *gambiae*, *A*. *arabiensis and A*. *rufipes*. PNA blocking in these species was compared with that observed for the V9 variable region of *A*. *coluzzii*.

### Amplification of hypervariable regions, V4 and V9, of the 18s rRNA gene

#### Amplification with annealing blocker

To test the annealing blockers, we evaluated the following variables:

(1) Variable region (V4 or V9) and (2) Annealing blockers (combined *Anopheles* and mammal blockers (0.8 μM each, 1.6 μM total).

For these tests, we used the following PCR recipe and cycling conditions: 3 μl template DNA, 0.6 μl 10x Qiagen PCR buffer, 0.24 μl MgCl_2_ (25 mM), 0.048 μl dNTP mix (25 mM), 0.3 μl DMSO, 0.003 μl 1000x SYBR Green, 0.12 μl ROX (25 μM), 0.03 μl Qiagen HotStarTaq Plus Polymerase, 0.3 μl forward primer (10 μM), 0.3 μl reverse primer (10 μM), 0.096 μl total volume of blocking primer(s) (100 μM), and 0.963 μl nuclease-free water. Samples were amplified on an ABI7900 thermocycler in 384-well plates using the following PCR cycling conditions: 95 °C, 15 minutes, and 25 cycles of: 94 °C, 30 seconds, 65 °C, 15 seconds, 55 °C, 30 seconds, and 72 °C, 30 seconds followed by a final extension of 72 °C 10 minutes. Samples were indexed and prepared for sequencing as described below.

#### Amplification with PNA blocker

Blocking of plant mitochondrial and chloroplast amplification by PNA oligonucleotides was shown to saturate at a concentration of 0.75 μM of PNA blocker^36^. However, in pilot experiments, we observed relatively small reductions in mosquito reads with 0.75 μM of PNA blocker, particularly in the case of the V9 region. Thus, we tested a range of concentrations of PNA blockers (0.75 μM, 1.5 μM, 3.75 μM, and 7.5 μM).

Samples were amplified using the following PCR recipe: 3 μl template DNA, 1.2 μl 5x KAPA HiFi buffer, 0.18 μl dNTP mix (10 mM), 0.3 μl DMSO, 0.003 μl 1000x SYBR Green, 0.12 μl ROX (25 μM), 0.06 μl KAPA HiFi HotStart Polymerase (Kapa Biosystems), 0.3 μl forward primer (10 μM), 0.3 μl reverse primer (10 μM), 0.045 μl of 100 μM PNA (1x condition, for other PNA conditions, the amount of PNA added was increased proportionately), nuclease-free water up to a reaction volume of 6 μl. The V9 primers amplified more efficiently than V4, a 1:5 dilution of template DNA was used for V9 amplifications, such that both samples underwent an approximately similar amount of amplification. PNAs were incubated at 55 °C for 5 minutes and vortexed to fully resuspend prior to adding to the reactions. Reactions were transferred into a 384-well plate and amplified with an ABI7900 thermocycler with the following amplification conditions: 95 °C, 5 minutes and 25 cycles of: 98 °C, 20 seconds, 78 °C, 5 seconds, 55 °C, 15 seconds, 72 °C, 1 minute. PCR products were diluted 1:100 in nuclease free water, and indexed using the procedure below.

#### Library construction from amplified products

Indexing PCRs were done using the following recipe: 5 µl template DNA, 1 µl nuclease-free water, 2 µl 5x KAPA HiFi buffer, 0.3 µl 10 mM dNTPs 0.5 µl DMSO, 0.2 µl KAPA HiFi Polymerase, 0.5 µl forward primer (10 µM), and 0.5 µl reverse primer (10 µM). Indexing PCR reactions were carried out in 96-well plates on a Bio-Rad Tetrad 2 thermocycler, using the following cycling conditions: 95 °C, 5 minutes and 10 cycles of: 98 °C, 20 seconds, 55 °C, 15 seconds, 72 °C, 1 minute, 72 °C, 10 minutes. The following indexing primers were used (X indicates the positions of 8 nucleotide unique indices for demultiplexing): Forward indexing primer: AATGATACGGCGACCACCGAGATCTACACXXXXXXXXTCGTCGGCAGCGTC Reverse indexing primer: CAAGCAGAAGACGGCATACGAGATXXXXXXXXGTCTCGTGGGCTCGG.

### Library normalization, pooling, and quantification

For the annealing blocker experiments, samples were normalized by quantifying the indexing PCR reactions using a Quant-iT PicoGreen dsDNA Assay Kit (ThermoFisher Scientific), normalizing samples for each amplicon (V4 and V9) to an equal concentration, pooling 3 µl of each normalized sample, purifying and concentrating with a 1x AmPureXP (Beckman Coulter) clean up, and eluting in 25 µl of Qiagen buffer EB. For the PNA blocker experiments, indexing PCR reactions were purified and normalized using a SequalPrep Normalization Plate Kit (ThermoFisher Scientific). 10 µl of each sample was pooled (V4 and V9 were pooled separately, due to the different sizes of these amplicons) and the pools were purified and concentrated with a 1x AmPureXP (Beckman Coulter) clean up, followed by elution in 25 µl of Qiagen buffer EB.

The concentrations of the amplicon pools were determined using a Quant-iT PicoGreen dsDNA Assay Kit (ThermoFisher Scientific) and amplicon sizes were verified on an Agilent Bioanalyzer High Sensitivity Chip. The V4 and V9 amplicon pools were independently diluted down to a 2 nM concentration in Qiagen EB buffer, and mixed at a 1:1 ratio.

### Library denaturation, dilution and sequencing

10 µl of the 2 nM sequencing library was denatured by adding 10 µl of 0.2 N NaOH and incubating at room temperature for 5 minutes, then the library was diluted to 8 pM in Illumina’s HT1 buffer, spiked with 15% PhiX, and sequenced on a portion of a MiSeq 2 × 300 (600 cycle v3) lane.

### Sequence analysis

Paired-end reads from MiSeq sequencing were quality trimmed and joined with Pandaseq^59^. Joined amplicons were clustered into Operational Taxonomic Units (OTUs) at a 97% similarity level using the pick_open_reference_otus.py script of QIIME version 1.9.1^60^. This script carries out an open-reference OTU picking strategy by first comparing against the 18s subdivision of the 119 release of Silva database and secondly clustering remaining sequences *de novo* ^61^. Only those OTUs containing more than 5 sequences were retained for subsequent analysis. Taxonomic assignment to the final OTUs was carried out with assign_taxonomy.py script of QIIME using UCLUST as classifier against the 18s subdivision of 119 release of Silva database. Rarefaction, alpha diversity, and OTU frequency changes were compared across samples and analyzed with QIIME and PhyloSeq^62^.

To compare the two blocking strategies (annealing blockers and PNA blockers) for species diversity and richness, an alpha-diversity analysis was carried out over 100 subsamplings of size 1000 of the initial datasets, over which the average number of observed species (richness) and the Shannon index (evenness) were estimated for each trial using the Phyloseq R package^62^. This approach estimated the species abundance of the real population while standardizing sampling effort. In order to evaluate if different concentrations of PNA blocker affect the composition of the eukaryotic microbiome in larval samples, mosquito and mammal OTUs were excluded from the OTU table, and chi-square tests were carried out over the filtered OTU table with the chisq.test function in R. Chi-square tests were carried out at different levels of the taxonomic classification according with UCLUST results.

## Electronic supplementary material

Supplementary Info
