# Supplementary material for: Preferential suppression of Anopheles gambiae host sequences allows detection of the mosquito eukaryotic microbiome
Source: Sci Rep. 2017 Jun 12;7:3241. doi: 10.1038/s41598-017-03487-1 (PMC5468309; doi:10.1038/s41598-017-03487-1)
Supplement: Supplementary file 1 — Supplementary Info [file 41598_2017_3487_MOESM1_ESM.pdf]

Preferential suppression of *Anopheles gambiae* host sequences allows detection of the mosquito eukaryotic microbiome

Eugeni Belda[1,2], Boubacar Coulibaly[3], Abdrahamane Fofana[3], Abdoul H. Beavogui[4], Sekou F. Traore[3], Daryl M. Gohl[5], Kenneth D. Vernick[1,2,*], Michelle M. Riehle[6]

[1]Department of Parasites and Insect Vectors, Unit of Genetics and Genomics of Insect Vectors, Institut Pasteur, Paris, France.
[2]CNRS Unit of Hosts, Vectors and Pathogens (URA3012), Paris, France
[3]Malaria Research and Training Centre (MRTC), Faculty of Medicine and Dentistry, University of Mali, Bamako, Mali.
[4]Centre de Formation et de Recherche en Santé Rurale de Mafèrinyah, Conakry, Republic of Guinea.
[5]University of Minnesota Genomics Center, Minneapolis, Minnesota, USA
[6]Department of Microbiology and Immunology, University of Minnesota, Minneapolis, MN, USA.

* Correspondence:
email: kvernick@pasteur.fr
address: Institut Pasteur, GGIV, 28 rue du Dr Roux, 75724 Paris Cedex 15, FRANCE

Supplementary Figure Legends

Figure S1: Rarefaction plots based on phylogenetic diversity. Samples were grouped according to their amplification with and without PNA (for PNA samples, data from all 4 PNA concentrations are pooled in this analysis). Phylogenetic diversity was computed from rarified OTU tables at sampling depths from 10 to 1000 sequences. At each sampling depth, the average and standard error of 10 rarified OTU tables is represented.

Figure S2: Taxonomic profile of larval sample from 18s rRNA gene amplicons of V4 and V9 hypervariable regions amplified at different concentrations of PNA blocker after exclusion of mosquito and mammalian sequences. Chi-square tests were carried out to test whether the presence or concentration of PNA blocker affects the relative fractions of the eukaryotic lineages detected. There was no significant difference in the composition of eukaryotic microbiota (chi-square p=0.92 for V4 and p=1 for V9) across all samples, with and without PNA blocker, showing that the presence of PNA blockers does not influence differential amplification of any particular eukaryotic microbe, and instead functions solely to block the amplification of the mosquito host template. Note that this Figure, as compared to Figures 3 and 4, provides the full taxonomic list of the larval sample only, with the Other category uncollapsed to show all lineages. The larval sample yielded most of the microbial diversity, and for simplicity the other two samples represented in Figures 3 and 4, Animal Bloodmeal and Human Bloodmeal, are not included here, so some classes in Figures 3 and 4 are not represented in Figure S4.

Figure S3: Multiple sequence alignment of representative 18s rRNA gene sequences from mosquito and mammal (Silva 119 database) with *Mammalia* and *Anopheles* anneal-inhibiting blocking primers targeting the V4 hypervariable region used in the present study. The alignment region corresponds to the 5’ end of the V4 hypervariable region.

Figure S4: Multiple sequence alignment of representative 18s rRNA gene sequences from mosquito and mammal (Silva 119 database) with *Mammalia* and *Anopheles* anneal-inhibiting blocking primers targeting the V9 hypervariable region used in the present study. The alignment region corresponds to the 3' end of the V9 hypervariable region.

Figure S5: Multiple sequence alignment of the 18s rRNA gene V9 hypervariable region of wild *A. coluzzii* (Acol) and *A. gambiae* (Agam) from Burkina Faso. Alignments represent the reversed and complemented genome region between nucleotide coordinates X:23669218-23669319 of the Vectorbase AgamP4 annotation. Sequence of the V9 PNA blocker (as shown in Table S1) is highlighted in purple.

Figure S6: Multiple sequence alignments as in Figure S5, but for the V4 hypervariable region. Alignments represent the genome region between nucleotide coordinates X:24339450-24339510 of the Vectorbase AgamP4 annotation. Sequence of the V4 PNA blocker (as shown in Table S1) is highlighted in purple.

Figure S1

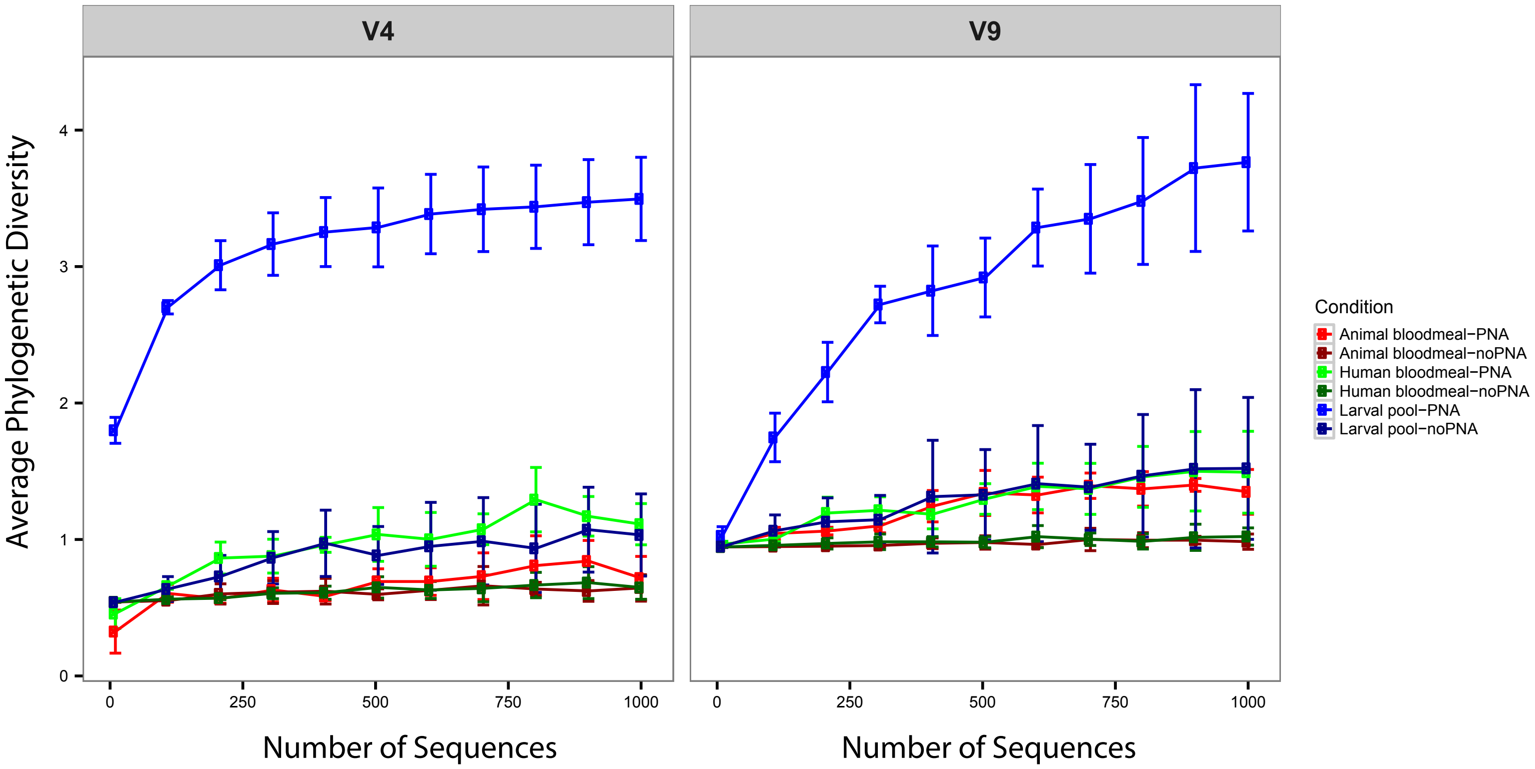
V4
V9
Average Phylogenetic Diversity
0
1
2
3
4
0
250
500
750
1000
Number of Sequences
Condition
Animal bloodmeal−PNA
Animal bloodmeal−noPNA
Human bloodmeal−PNA
Human bloodmeal−noPNA
Larval pool−PNA
Larval pool−noPNA

Figure S2

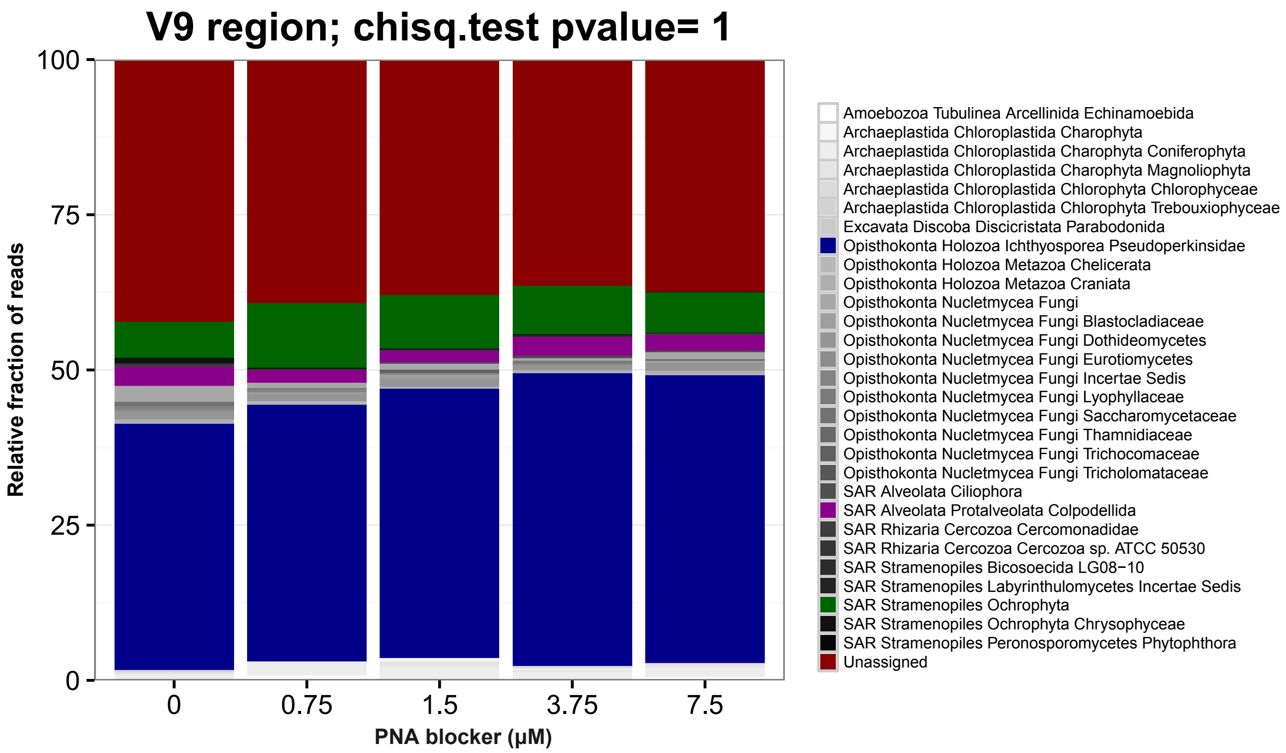
V9 region; chisq.test pvalue= 1
100
75
50
25
0
Relative fraction of reads
0
0.75
1.5
3.75
7.5
PNA blocker (μM)
Amoebozoa Tubulinea Arcellinida Echinamoebida
Archaeplastida Chloroplastida Charophyta
Archaeplastida Chloroplastida Charophyta Coniferophyta
Archaeplastida Chloroplastida Charophyta Magnoliophyta
Archaeplastida Chloroplastida Chlorophyta Chlorophyceae
Archaeplastida Chloroplastida Chlorophyta Trebouxiophyceae
Excavata Discoba Discicristata Parabodonida
Opisthokonta Holozoa Ichthyosporea Pseudoperkinsidae
Opisthokonta Holozoa Metazoa Chelicerata
Opisthokonta Holozoa Metazoa Craniata
Opisthokonta Nucletmycea Fungi
Opisthokonta Nucletmycea Fungi Blastocladiaceae
Opisthokonta Nucletmycea Fungi Dothideomycetes
Opisthokonta Nucletmycea Fungi Eurotiomycetes
Opisthokonta Nucletmycea Fungi Incertae Sedis
Opisthokonta Nucletmycea Fungi Lyophyllaceae
Opisthokonta Nucletmycea Fungi Saccharomycetaceae
Opisthokonta Nucletmycea Fungi Thamnidiaceae
Opisthokonta Nucletmycea Fungi Trichocomaceae
Opisthokonta Nucletmycea Fungi Tricholomataceae
SAR Alveolata Ciliophora
SAR Alveolata Protalveolata Colpodellida
SAR Rhizaria Cercozoa Cercomonadidae
SAR Rhizaria Cercozoa Cercozoa sp. ATCC 50530
SAR Stramenopiles Bicosoecida LG08−10
SAR Stramenopiles Labyrinthulomycetes Incertae Sedis
SAR Stramenopiles Ochrophyta
SAR Stramenopiles Ochrophyta Chrysophyceae
SAR Stramenopiles Peronosporomycetes Phytophthora
Unassigned

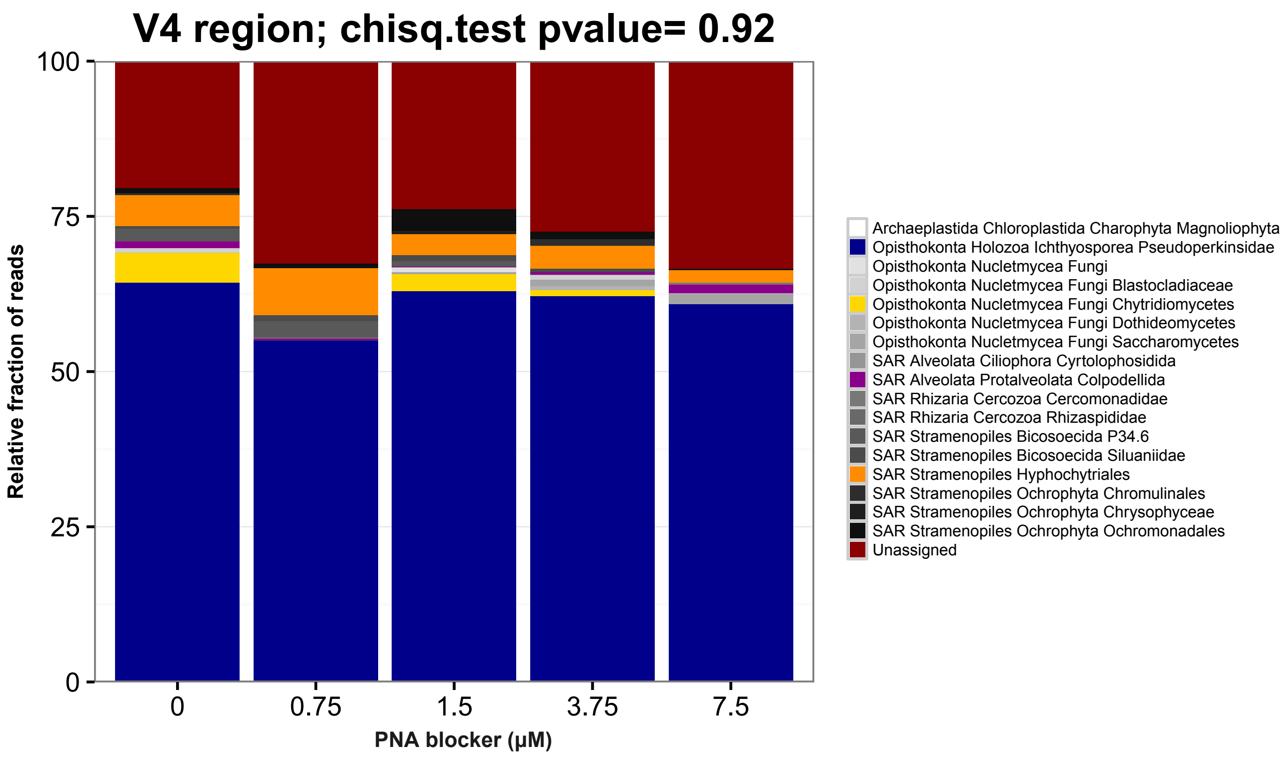
V4 region; chisq.test pvalue= 0.92
100
75
50
25
0
Relative fraction of reads
0
0.75
1.5
3.75
7.5
PNA blocker (μM)
Archaeplastida Chloroplastida Charophyta Magnoliophyta
Opisthokonta Holozoa Ichthyosporea Pseudoperkinsidae
Opisthokonta Nucletmycea Fungi
Opisthokonta Nucletmycea Fungi Blastocladiaceae
Opisthokonta Nucletmycea Fungi Chytridiomycetes
Opisthokonta Nucletmycea Fungi Dothideomycetes
Opisthokonta Nucletmycea Fungi Saccharomycetes
SAR Alveolata Ciliophora Cyrtolophosidida
SAR Alveolata Protalveolata Colpodellida
SAR Rhizaria Cercozoa Cercomonadidae
SAR Rhizaria Cercozoa Rhizaspididae
SAR Stramenopiles Bicosoecida P34.6
SAR Stramenopiles Bicosoecida Siluaniidae
SAR Stramenopiles Hyphochytriales
SAR Stramenopiles Ochrophyta Chromulinales
SAR Stramenopiles Ochrophyta Chrysophyceae
SAR Stramenopiles Ochrophyta Ochromonadales
Unassigned

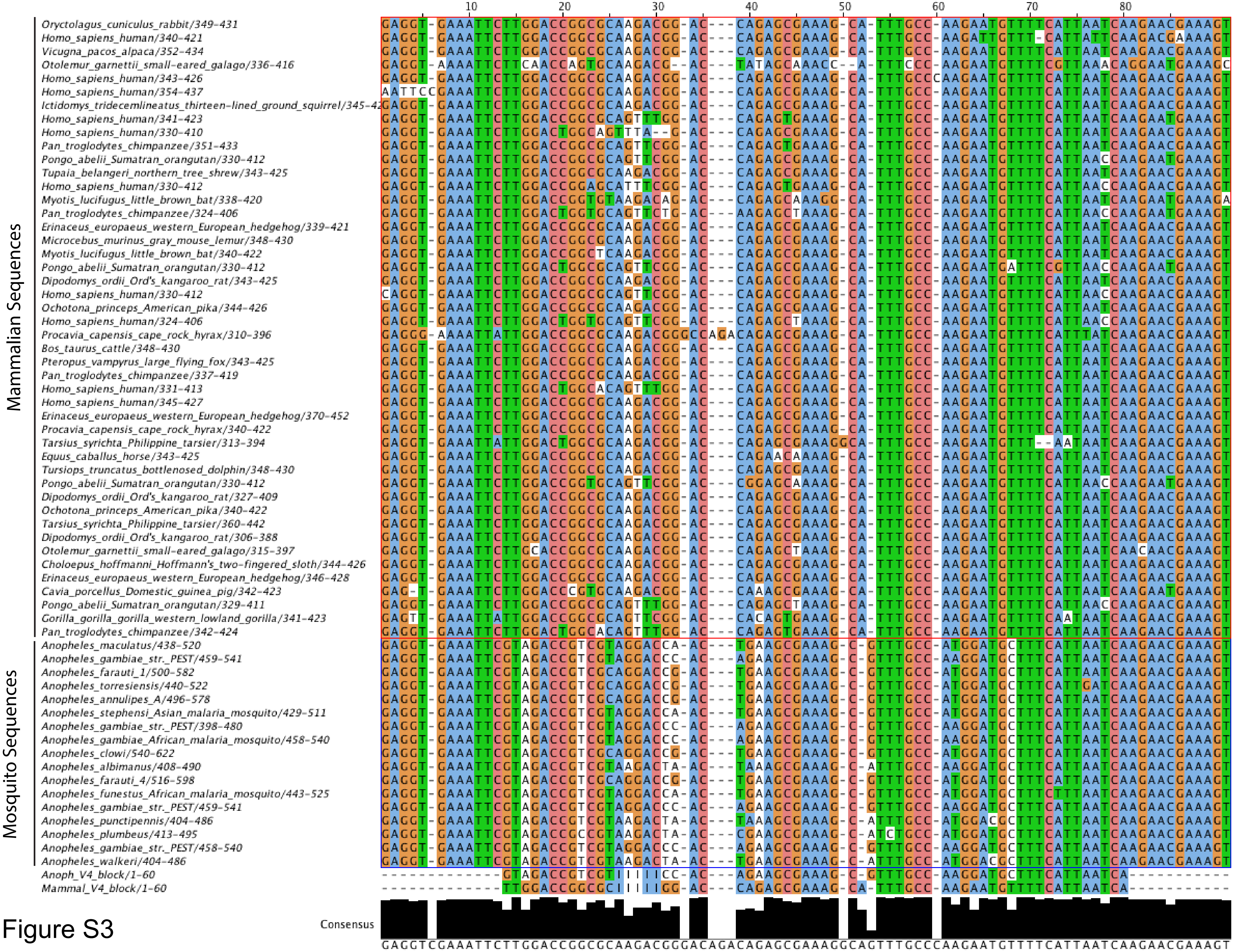
10
20
30
40
50
60
70
80
Mammalian Sequences
Oryctolagus_cuniculus_rabbit/349-431
Homo_sapiens_human/340-421
Vicugna_pacos_alpaca/352-434
Otolemur_garnettii_small-eared_galago/336-416
Homo_sapiens_human/343-426
Homo_sapiens_human/354-437
Ictidomys_tridecemlineatus_thirteen-lined_ground_squirrel/345-42
Homo_sapiens_human/341-423
Homo_sapiens_human/330-410
Pan_troglodytes_chimpanzee/351-433
Pongo_abelii_Sumatran_orangutan/330-412
Tupaia_belangeri_northern_tree_shrew/343-425
Homo_sapiens_human/330-412
Myotis_lucifugus_little_brown_bat/338-420
Pan_troglodytes_chimpanzee/324-406
Erinaceus_europaeus_western_European_hedgehog/339-421
Microcebus_murinus_gray_mouse_lemur/348-430
Myotis_lucifugus_little_brown_bat/340-422
Pongo_abelii_Sumatran_orangutan/330-412
Dipodomys_ordii_Ord's_kangaroo_rat/343-425
Homo_sapiens_human/330-412
Ochotona_princeps_American_pika/344-426
Homo_sapiens_human/324-406
Procavia_capensis_cape_rock_hyrax/310-396
Bos_taurus_cattle/348-430
Pteropus_vampyrus_large_flying_fox/343-425
Pan_troglodytes_chimpanzee/337-419
Homo_sapiens_human/331-413
Homo_sapiens_human/345-427
Erinaceus_europaeus_western_European_hedgehog/370-452
Procavia_capensis_cape_rock_hyrax/340-422
Tarsius_syrichta_Philippine_tarsier/313-394
Equus_caballus_horse/343-425
Tursiops_truncatus_bottlenosed_dolphin/348-430
Pongo_abelii_Sumatran_orangutan/330-412
Dipodomys_ordii_Ord's_kangaroo_rat/327-409
Ochotona_princeps_American_pika/340-422
Tarsius_syrichta_Philippine_tarsier/360-442
Dipodomys_ordii_Ord's_kangaroo_rat/306-388
Otolemur_garnettii_small-eared_galago/315-397
Choloepus_hoffmanni_Hoffmann's_two-fingered_sloth/344-426
Erinaceus_europaeus_western_European_hedgehog/346-428
Cavia_porcellus_Domestic_guinea_pig/342-423
Pongo_abelii_Sumatran_orangutan/329-411
Gorilla_gorilla_gorilla_western_lowland_gorilla/341-423
Pan_troglodytes_chimpanzee/342-424
Mosquito Sequences
Anopheles_maculatus/438-520
Anopheles_gambiae_str._PEST/459-541
Anopheles_farauti_1/500-582
Anopheles_torresiensis/440-522
Anopheles_annulipes_A/496-578
Anopheles_stephensi_Asian_malaria_mosquito/429-511
Anopheles_gambiae_str._PEST/398-480
Anopheles_gambiae_African_malaria_mosquito/458-540
Anopheles_clowi/540-622
Anopheles_albimanus/408-490
Anopheles_farauti_4/516-598
Anopheles_funestus_African_malaria_mosquito/443-525
Anopheles_gambiae_str._PEST/459-541
Anopheles_punctipennis/404-486
Anopheles_plumbeus/413-495
Anopheles_gambiae_str._PEST/458-540
Anopheles_walkeri/404-486
Anoph_V4_block/1-60
Mammal_V4_block/1-60
Consensus
GAGGTCGAAATTCTTGGACCGGCGCAAGACGGGACAGACAGAGCGAAAGGCAGTTTGCCCAAGAATGTTTTCATTAATCAAGAACGAAAGT

Figure S3

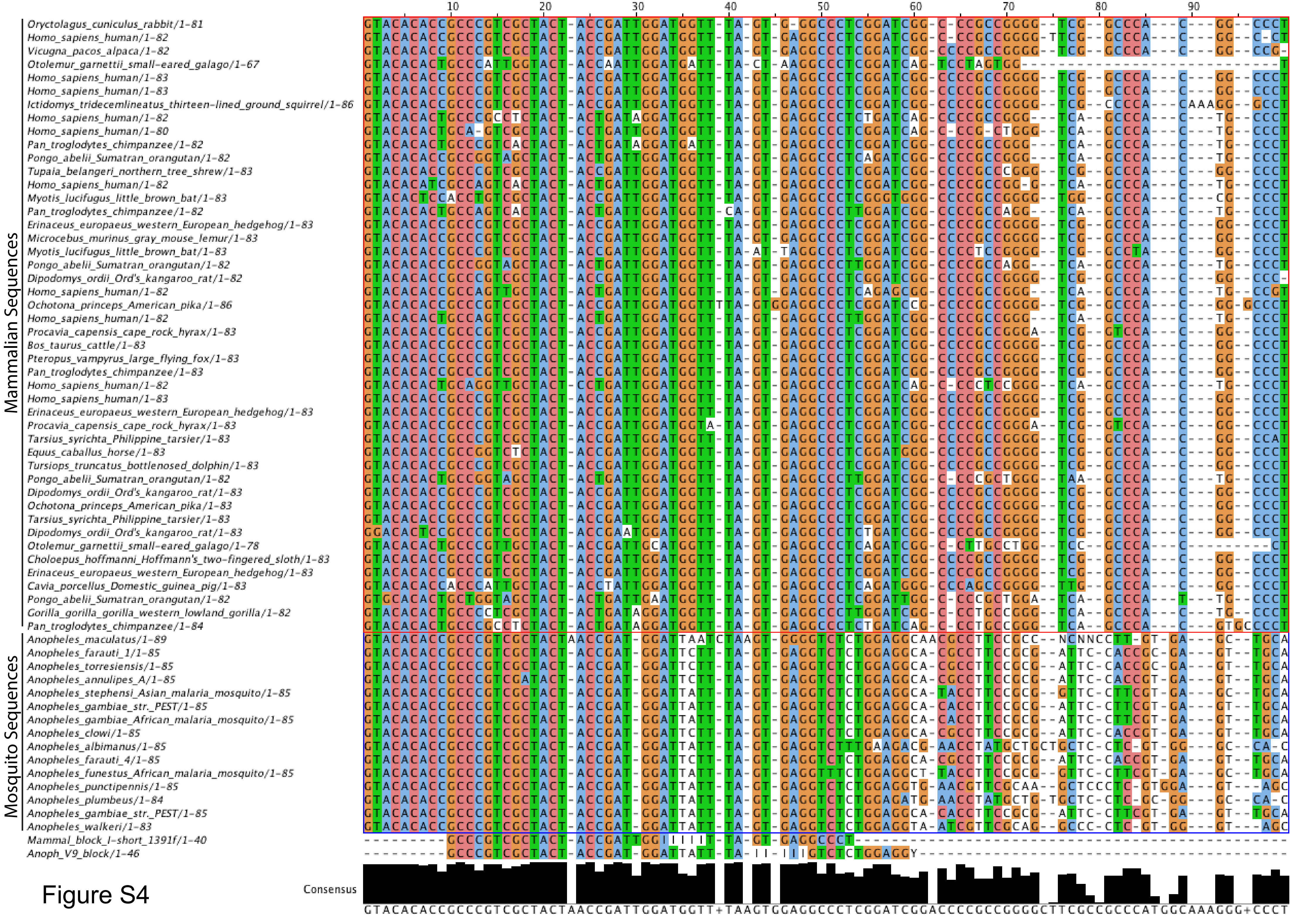

Figure S4

Figure S5

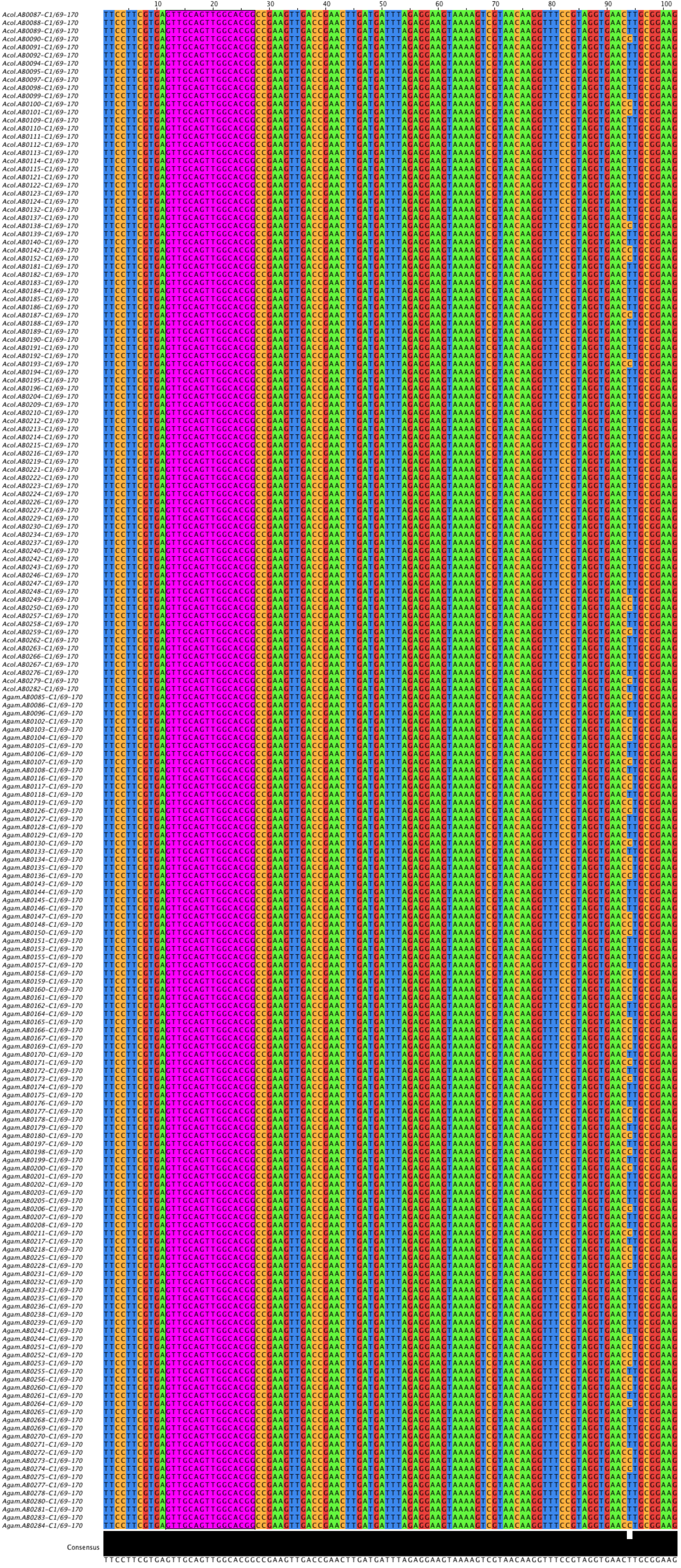

Figure S6

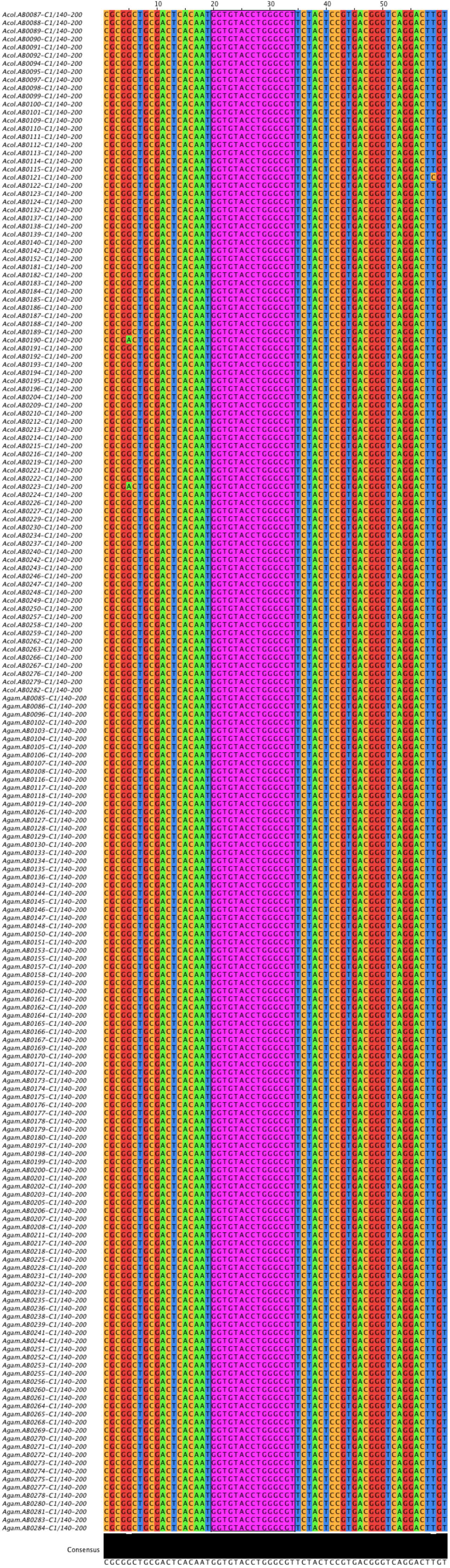
10
20
30
40
50
Consensus
CGCGGCTGCGACTCACAATGGTGTACCTGGCGTTCTACTCCGTGACGGTCAGGACTTGT

Table S1. Primers used for Anneal Blocking and PNA Blocking

Anneal Blockers

**Hypervariable Region**	**Name**	**Sequence (5'->3')**
V9	Mammal_block_I-short_1391f	GCCCGTCGCTACTACCGATTGG/ideoxyI//ideoxyI//ideoxyI//ideoxyI//ideoxyI/TTAGTGAGGCCCT/3SpC3/
	Anoph_V9_block	GCCCGTCGCTACTACCGATGGATTATTTA/ideoxyI//ideoxyI//ideoxyI//ideoxyI//ideoxyI/GTCTCTGGAGGY/3SpC3/
	18S_V9_1391_F_Nextera*	TCGTCGGCAGCGTCAGATGTGTATAAGAGACAG**GTACACACCGCCCGTC**
	18S_V9_EukBr_R_Nextera*	GTCTCGTGGGCTCGGAGATGTGTATAAGAGACAG**TGATCCTTCTGCAGGTTCACCTAC**
V4	Mammal_V4_block	TGATTAATGAAAACATTCTTGGCAAATGCTTTCGCTCTGGTCC/ideoxyI//ideoxyI//ideoxyI//ideoxyI//ideoxyI/GCGCCGGTCCAA/3SpC3/
	Anoph_V4_block	TGATTAATGAAAGCATCCTTGGCAAACGCTTTCGCTTCTGTGG/ideoxyI//ideoxyI//ideoxyI//ideoxyI//ideoxyI/ACGACGGTCTAC/3SpC3/
	18S_V4F_Nextera*	TCGTCGGCAGCGTCAGATGTGTATAAGAGACAG**CCAGCASCYGCGGTAATTCC**
	18S_V4R_Nextera*	GTCTCGTGGGCTCGGAGATGTGTATAAGAGACAG**ACTTTCGTTCTTGATYRA**

*The 18S targetting portions of the primer are in bold, adaptor tails for sequencing are in plain text

PNA Blockers

**Hypervariable Region**	**Name**	**Sequence (5'->3')**
V9	AgV9-PNA	CCGTGCCAACTGCAAC
V4	AgV4-PNA	ACGCCCAGGTACACC
**Hypervariable region 1**	**Forward primer***	**Reverse primer***
V9	TCGTCGGCAGCGTCAGATGTGTATAAGAGACAG**GTACACACCGCCCGTC**	GTCTCGTGGGCTCGGAGATGTGTATAAGAGACAG**TGATCCTTCTGCAGGTTCACCTAC**
V4	TCGTCGGCAGCGTCAGATGTGTATAAGAGACAG**CCAGCASCYGCGGTAATTCC**	GTCTCGTGGGCTCGGAGATGTGTATAAGAGACAG**ACTTTCGTTCTTGATYRA**

*The 18S targetting portions of the primer are in bold, adaptor tails for sequencing are in plain text
